# Renal Ischemia/Reperfusion Early Induces Myostatin and PCSK9 Expression in Rat Kidneys and HK-2 Cells

**DOI:** 10.3390/ijms22189884

**Published:** 2021-09-13

**Authors:** Chiara Barisione, Daniela Verzola, Silvano Garibaldi, Pier Francesco Ferrari, Giacomo Garibotto, Pietro Ameri, Bianca Pane, Giovanni Spinella, Giovanni Pratesi, Domenico Palombo

**Affiliations:** 1 Department of Surgical and Integrated Diagnostic Sciences, University of Genoa, Viale Benedetto XV 6, 16132 Genoa, Italy; chiara.barisione@unige.it (C.B.); bianca.pane@unige.it (B.P.); giovanni.spinella@unige.it (G.S.); giovanni.pratesi@unige.it (G.P.); domenico.palombo@unige.it (D.P.); 2 Department of Internal Medicine, University of Genoa, Viale Benedetto XV 6, 16132 Genoa, Italy; daniela.verzola@unige.it (D.V.); silvano.garibaldi@unige.it (S.G.); gari@unige.it (G.G.); pietro.ameri@unige.it (P.A.); 3 Division of Nephrology, Dialysis and Transplantation, IRCCS Ospedale Policlinico San Martino, Largo Rosanna Benzi 10, 16132 Genoa, Italy; 4 Cardiovascular Disease Unit, IRCCS Ospedale Policlinico San Martino, Largo Rosanna Benzi 10, 16132 Genoa, Italy; 5 Vascular and Endovascular Surgery Unit, IRCCS Ospedale Policlinico San Martino, Largo Rosanna Benzi 10, 16132 Genoa, Italy; 6 Research Center for Biologically Inspired Engineering in Vascular Medicine and Longevity, University of Genoa, Via Montallegro 1, 16145 Genoa, Italy

**Keywords:** oxidative stress, mitochondrial dysfunction, renal injury, aorta cross-clamping, tubular necrosis

## Abstract

During visceral interventions, the transient clampage of supraceliac aorta causes ischemia/reperfusion (I/R) in kidneys, sometime resulting in acute renal failure; preclinical studies identified redox imbalance as the main driver of I/R injury. However, in humans, the metabolic/inflammatory responses seem to prevail on oxidative stress. We investigated myostatin (Mstn) and proprotein convertase subtilisin/kexin type 9 (PCSK9), proatherogenic mediators, during renal I/R. Compared to sham-operated animals, the kidneys of rats who had experienced ischemia (30 min) had higher Mstn and PCSK9 expression after 4 h of reperfusion. After 24 h, they displayed tubular necrosis, increased nitrotyrosine positivity, and nuclear peroxisome proliferator-activated receptor gamma coactivator-1alpha relocation, markers of oxidative stress and mitochondria imbalance. Mstn immunopositivity was increased in tubuli, while PCSK9 immunosignal was depleted; systemically, PCSK9 was higher in plasma from I/R rats. In HK-2 cells, both ischemia and reperfusion enhanced reactive oxygen species production and mitochondrial dysfunction. H_2_O_2_ upregulated Mstn and PCSK9 mRNA after 1 and 3.5 h, respectively. Accordingly, ischemia early induced Mstn and PCSK9 mRNA; during reperfusion Mstn was augmented and PCSK9 decreased. Mstn treatment early increased PCSK9 expression (within 8 h), to diminish over time; finally, Mstn silencing restrained ischemia-induced PCSK9. Our study demonstrates that renal I/R enhances Mstn and PCSK9 expression and that Mstn induces PCSK9, suggesting them as therapeutic targets for vascular protection during visceral surgery.

## 1. Introduction

Visceral vascular surgery often requires aortic cross-clamping proximally to the celiac artery, causing rapid hemodynamic changes in the downstream organs: the sudden decrease of the blood flow and the acute hypoxic moiety drive the shift from aerobic to anaerobic metabolism, the development of acidosis, and the increased cell membrane permeability; then, tissue reperfusion after clamp removal leads to the activation of pro-oxidant, inflammatory pathways, and cellular damage. This sequence of events causes the so-called ischemia/reperfusion (I/R) injury in kidneys, liver, bowel, spinal cord, and inferior limbs.

The severity of the damage depends on the single tissue resistance based on its metabolic needs. Kidneys, which have an active metabolism and an extensively developed vascular bed, are the main target and I/R is often the underlying mechanism of the post-surgical acute kidney injury.

At a cellular level, the key organelles in I/R are mitochondria; specifically, ischemia leads to alterations of the mitochondrial electron transporter chain complexes, reduced efficacy of the antioxidant system, and the stimulation of anaerobic metabolism with the breakdown of ATP to ADP and AMP, the accumulation of lactic acid, and the decrease of mitochondrial pH. Reperfusion is characterized by the reintroduction of O_2_, which causes significant production of reactive oxygen species (ROS). The overproduced superoxide anion radical (O_2_^•−^) may react with nitric oxide to form peroxynitrite, followed by the nitration of proteins, the oxidation of thiols, or decomposition into hydroxyl radical (^•^OH).

In both phases, there is overproduction of reactive oxygen and nitrogen species (RONS), which affects proteins, lipids, and DNA integrity, leading to further impairment of mitochondrial function and also to cell death [1].

Previous preclinical studies focused mainly on oxidative damage and proposed the use of several compounds with antioxidant properties as protective strategies [2,3,4,5,6].

However, the available clinical trials demonstrated that merely antioxidant preventive approaches are poorly effective in humans, where metabolic dysfunctions seem to prevail on ROS damage following I/R injury [7].

Several mediators of inflammation and vascular diseases have been demonstrated to interact with metabolic pathways and promising results have been obtained employing antidiabetic treatments in an experimental animal model of I/R [8].

Recently, I/R injury in the heart has been associated with overexpression of two metabolic mediators, proprotein convertase subtilisin/kexin type 9 (PCSK9) and myostatin (Mstn).

PCSK9, a pivotal regulator of low-density lipoprotein cholesterol (LDL-C), negatively affects infarct district and cardiac function [9]. Preclinical studies demonstrated that PCSK9 inhibition ameliorates infarct size and recovery after myocardial I/R injury [10]. PCSK9 is mainly expressed in the liver, but the kidney is also an important extra-hepatic source.

Mstn is a TGF-β superfamily member with anti-anabolic function; a known playmaker in cachexia and sarcopenia, it has been found upregulated in skeletal muscle cells exposed to oxidative stress conditions in the context of chronic obstructive pulmonary disease [11]. Mstn is also highly expressed 12 h after myocardial infarction in human hearts and 10 min after ischemia in the heart and blood of mouse, where it contributed to skeletal muscle atrophy by upregulating atrogin and muscle RING-finger protein-1 (MuRF-1) expression [12].

We previously recognized Mstn as a mediator of atherosclerosis progression and vascular inflammation [13]; more recently, Mstn enhancement has been observed also in the tubulointerstitial compartment of the human kidney during diabetic nephropathy, suggesting its contribution to renal interstitial fibrosis [14].

In order to identify new targets for protection from renal I/R injury, in this work we first evaluated the renal redox profile and the Mstn and PCSK9 expression in the kidneys of Sprague Dawley rats that had undergone surgically-induced I/R. Then, using an in vitro model of I/R, we investigated the mitochondrial function, redox imbalance, the expression of Mstn and PCSK9, and their interplay in tubular renal HK-2 cells.

## 2. Results

### 2.1. I/R Induces Mstn and PCSK9 Protein Expression in Rat Kidneys 4 h after Reflow

**Figure 1:**
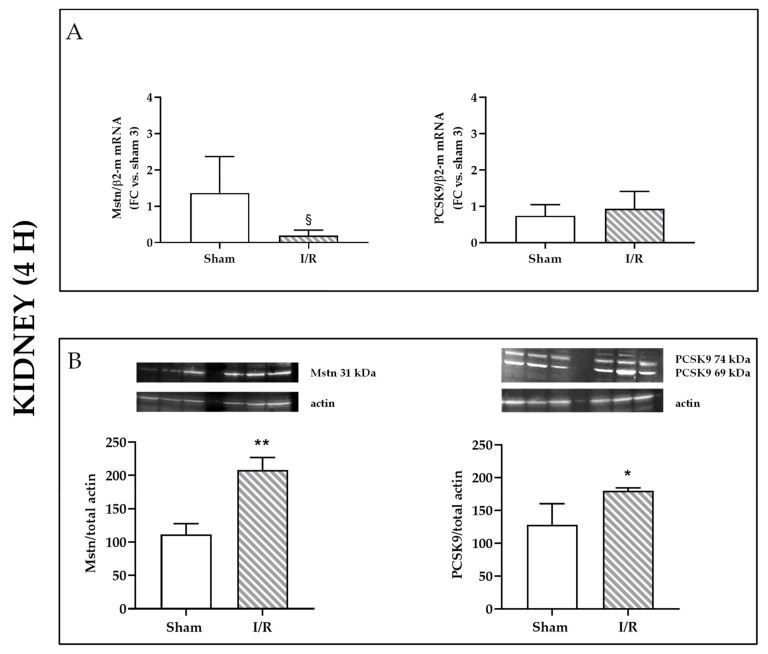
Analysis of rat kidney homogenates after sham intervention or ischemia/reperfusion (I/R, 30 min/4 h). (**A**) Mstn and PCSK9 mRNA expression by quantitative real-time PCR. The results have been normalized by expressing the number of transcript copies as a ratio to β2-microglobulin (β2-m) and indicated as fold changes (FC) in respect to kidneys from sham-operated rat (#3) (§ *p* = 0.05, one tailed analysis). (**B**) Western blot analysis of Mstn 31 kDa (left) and of PCSK9 74 and 69 kDa isoforms (right). Bands of interest were normalized against those obtained by membrane rehybridization with anti-total actin antibody. Representative immunoblots are shown. Data are expressed as % ± SEM in respect to sham-operated rat (#1); * *p* < 0.05, ** *p* < 0.01.

Kidneys of Sprague Dawley rats who had undergone 30 min of ischemia revealed, after 4 h of reflow, changes in Mstn and PCSK9: although mRNA levels were not significantly affected, with a tendency to Mstn lowering mRNA (Figure 1A), protein expression was augmented for both Mstn and PCSK9 (*p* < 0.01 and *p* < 0.05, respectively), as detected by western blot analysis (Figure 1B).

At this time point, no changes for monocyte chemoattractant protein-1 (MCP-1) mRNA have been observed in kidney homogenates from I/R and sham-operated rats (Supplementary Figure S1).

### 2.2. I/R Induces Focal Tubular Necrosis, Oxidative Stress, Peroxisome Proliferator-Activated Receptor Gamma Coactivator-1alpha (PGC-1α) Activation and Increased MCP-1 Gene Expression in Rat Kidneys 24 h after Reflow

**Figure 2:**
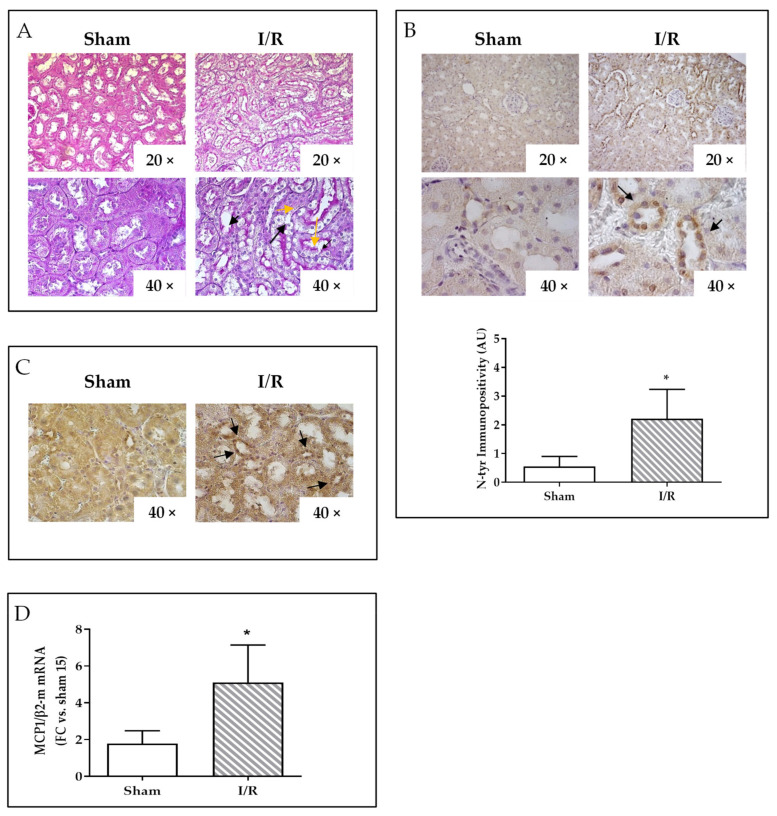
Analysis of rat kidneys after sham intervention or ischemia/reperfusion (I/R, 30 min/24 h). (**A**) PAS staining showing areas of tubular necrosis in I/R kidney (right panels): yellow arrows indicate swollen cells and black arrows indicate the site of cell loss within the tubuli. (**B**) N-tyr immunostaining, representative images (top) and image analysis quantification (bottom); * *p* < 0.05. (**C**) PGC-1α immunostaining: black arrows display the nuclear positivity in representative images. (**D**) MCP-1 mRNA expression after 24 h of reperfusion by quantitative real-time PCR. The results have been normalized by expressing the number of transcript copies as a ratio to β2-microglobulin (β2-m) and indicated as fold changes (FC) in respect to kidneys from sham-operated rat (#15); * *p* < 0.05.

After 24 h of reflow, kidneys of rats displayed typical features of tubular focal necrosis [15]. Periodic acid-Schiff (PAS) histological staining revealed a patch-like distribution of viable and damaged tubular cells; necrotic cells were swollen, with unevenly stained cytoplasm, and some of them were sloughed into the tubuli lumen (Figure 2A).

Immunopositivity for nitrotyrosine (N-tyr), a marker of reactive oxygen species (ROS) accumulation, was significantly increased (about three fold, *p* < 0.05) by I/R in tubular cells, both in the cytoplasms and nuclei (Figure 2B); immunostaining for the PGC-1α, a transcriptional coactivator regulating genes involved in energy metabolism and mitochondrial biogenesis, differed for the intracellular distribution: in the cytoplasmatic compartment under basal condition, it localized in the nucleus of tubular cells upon I/R (Figure 2C), indicating a signal activation in the attempt to obtain mitochondrial function homeostasis.

Renal tissue homogenates of I/R rats revealed a 3.8 fold increase for MCP-1 mRNA in comparison to kidneys from sham-operated animals (*p* < 0.05) (Figure 2D).

### 2.3. Effects of I/R on Mstn and PCSK9 in Rat Kidneys and on PCSK9 in Rat Plasma 24 h after Reflow

**Figure 3:**
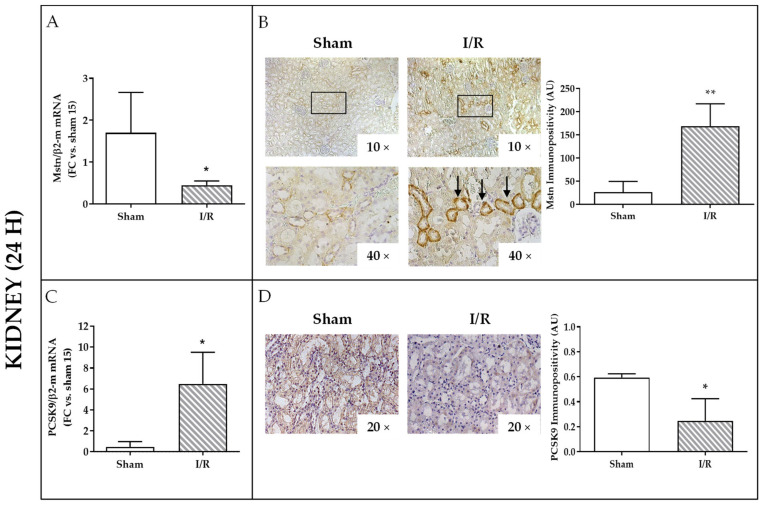

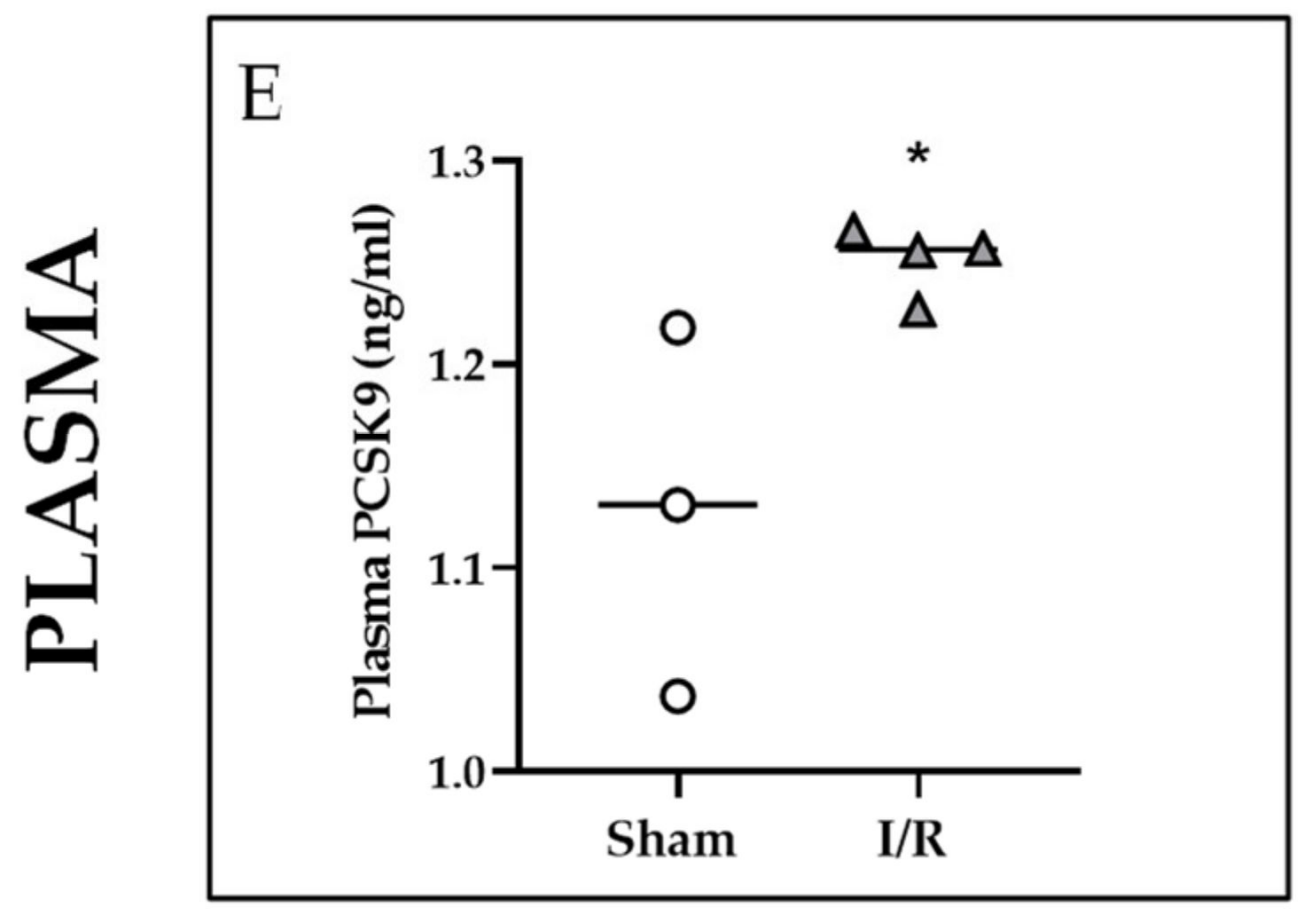
Analysis of Mstn and PCSK9 after sham intervention or ischemia/reperfusion (I/R, 30 min/24 h) in kidneys. (**A**) Mstn mRNA expression by quantitative real-time PCR. The results have been normalized by expressing the number of transcript copies as a ratio to β2-microglobulin (β2-m) and indicated as fold changes (FC) in respect to kidneys from sham-operated rat (#15) (left); * *p* < 0.05. (**B**) Immunostaining: in representative images, arrows indicate sites of intense positivity in tubular cells (left); image analysis quantification (right); ** *p* < 0.01. (**C**) PCSK9 mRNA expression by quantitative real-time PCR. The results have been normalized by expressing the number of transcript copies as a ratio to β2-microglobulin (β2-m) and indicated as fold changes (FC) in comparison to kidneys from sham-operated rat (#15) (left); * *p* < 0.05. (**D**) Representative images of immunohistochemistry (left) and image analysis quantification (right); * *p* < 0.05. (**E**) PCSK9 level in rat plasma by enzyme linked immunosorbent assay; * *p* < 0.05.

We next explored Mstn and PCSK 9 expression in the kidneys of rats that underwent ischemia after 24 h of reflow. In whole tissue homogenates, Mstn mRNA was reduced (3.6 fold decrease versus sham-operated animals, *p* < 0.05) (Figure 3A); in contrast, immunopositivity was sensibly augmented of about six fold (*p* < 0.01) and was mainly localized in tubular cells (Figure 3B). PCSK9 mRNA was highly upregulated (12 fold increase versus sham-operated animals; *p* < 0.05) (Figure 3C); by contrast, immunostaining displayed a solid PCSK9 depletion from tubular cells, halving the signal intensity in comparison to kidneys from sham-operated rats (*p* < 0.05) (Figure 3D). Systemically, a slight but significant increase of PCSK9 was observed in plasma from I/R rats (*p* < 0.05) (Figure 3E), while creatinine levels, enhanced at the previous time point (4 h), 24 h after reflow did not differ between sham-operated and I/R animals (Supplementary Figure S2).

### 2.4. Effects of I/R on Cell Cycle and Oxidative Stress in HK-2 Renal Tubular Cells

In order to dissect the complex cascade of cell events early induced by I/R in vivo, we analyzed in vitro the effects of I/R-like conditions on cycle progression, oxidative stress, and mitochondrial balance in HK-2 human tubular cells. Cells were first incubated 20 h in an anoxic chamber and in a serum free culture medium to resemble ischemia, then exposed again to complete medium and normoxia for further 3 or 24 h (reperfusion-like condition).

**Figure 4:**
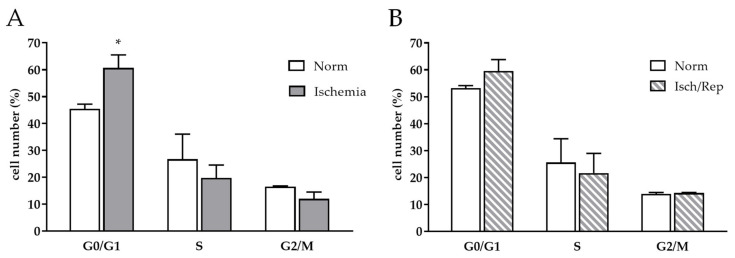

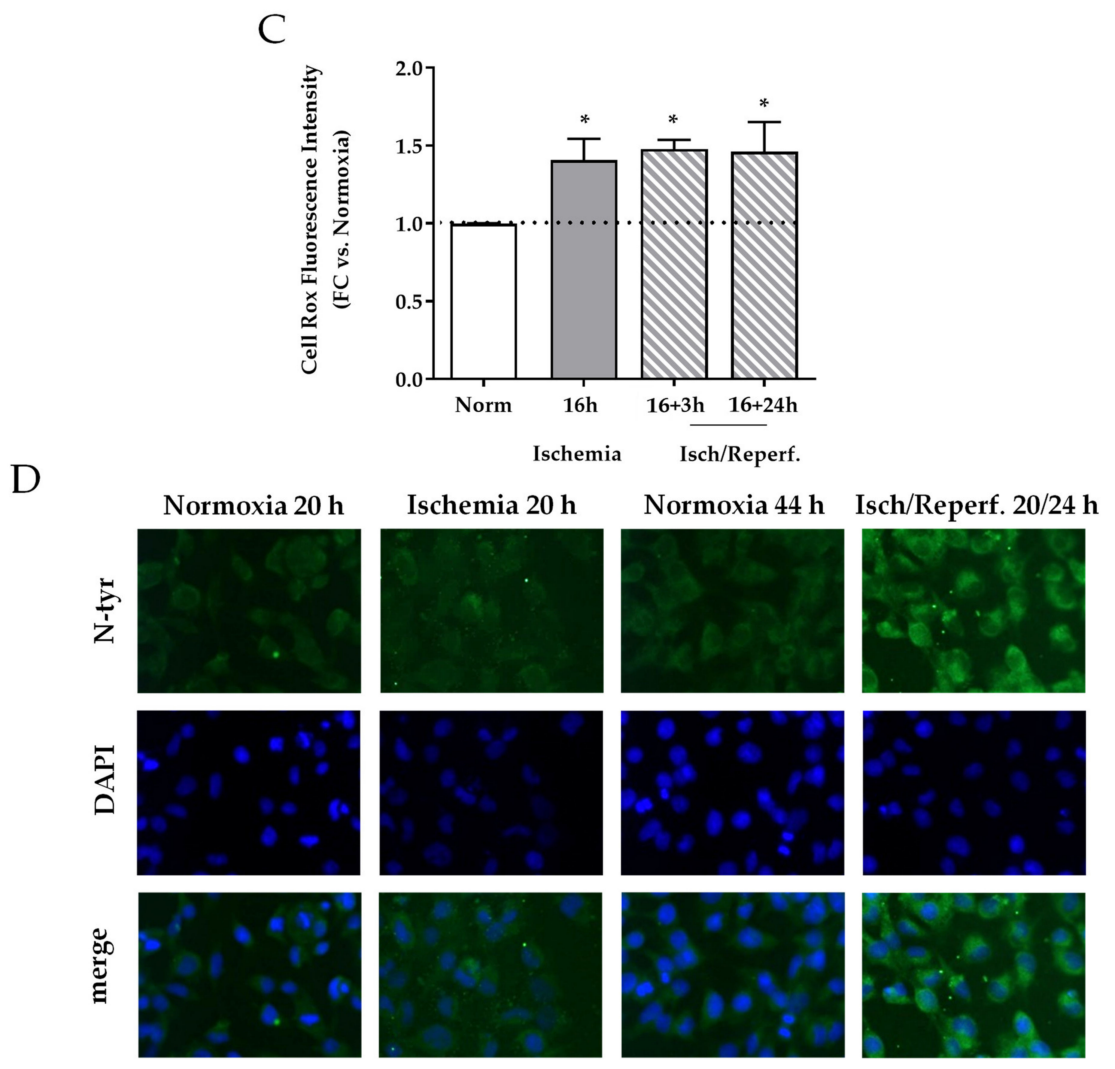
Cell cycle distribution and oxidative stress during I/R in HK-2 tubular renal cells. Flow cytometry analysis of cell cycle phases determined after (**A**) 20 h of ischemia and (**B**) 24 h of reperfusion using the Triton/propidium iodide solution; * *p* < 0.05. (**C**) Quantification of cell ROS production after 16 h of ischemia and 3–24 h of reperfusion using the CellROX™ kit; * *p* < 0.05. (**D**) Immunofluorescence for N-tyr, green signal (Alexa 488).

Looking at the cell cycle progression, 20 h of ischemia promoted an increase in the G0/G1 phase (*p* < 0.05), while no significant difference was recorded in the S and G2/M phase when compared to cells kept in standard cell culture conditions (“Normoxia”) (*p* < 0.05) (Figure 4A). A further 24 h of normoxia and complete culture medium restored the proliferative rate at the same extent as normoxic control cells (Figure 4B).

ROS production was measured with the CellROX™ probe after 16 h of ischemia and 3 and 24 h after having restored reperfusion-like conditions. A 50% increase of intracellular ROS was recorded at each time point (*p* < 0.05) (Figure 4C). Accordingly, an immunofluorescence signal for N-tyr, a stable marker for oxidative damage, was slightly increased after ischemia and highly enhanced 24 h after reperfusion (Figure 4D).

### 2.5. Effects of I/R on Mitochondria in HK-2 Renal Tubular Cells

**Figure 5:**
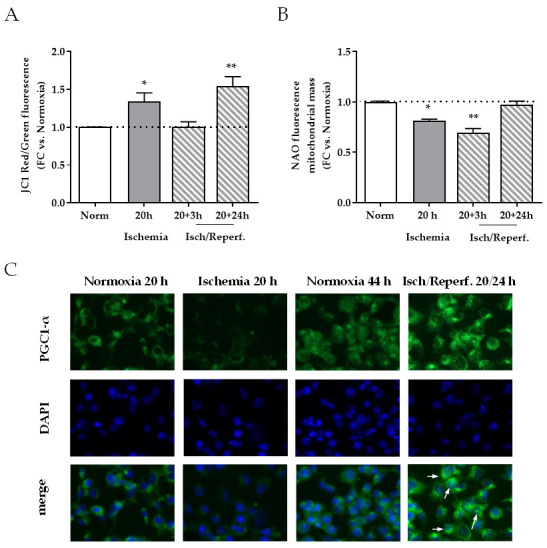

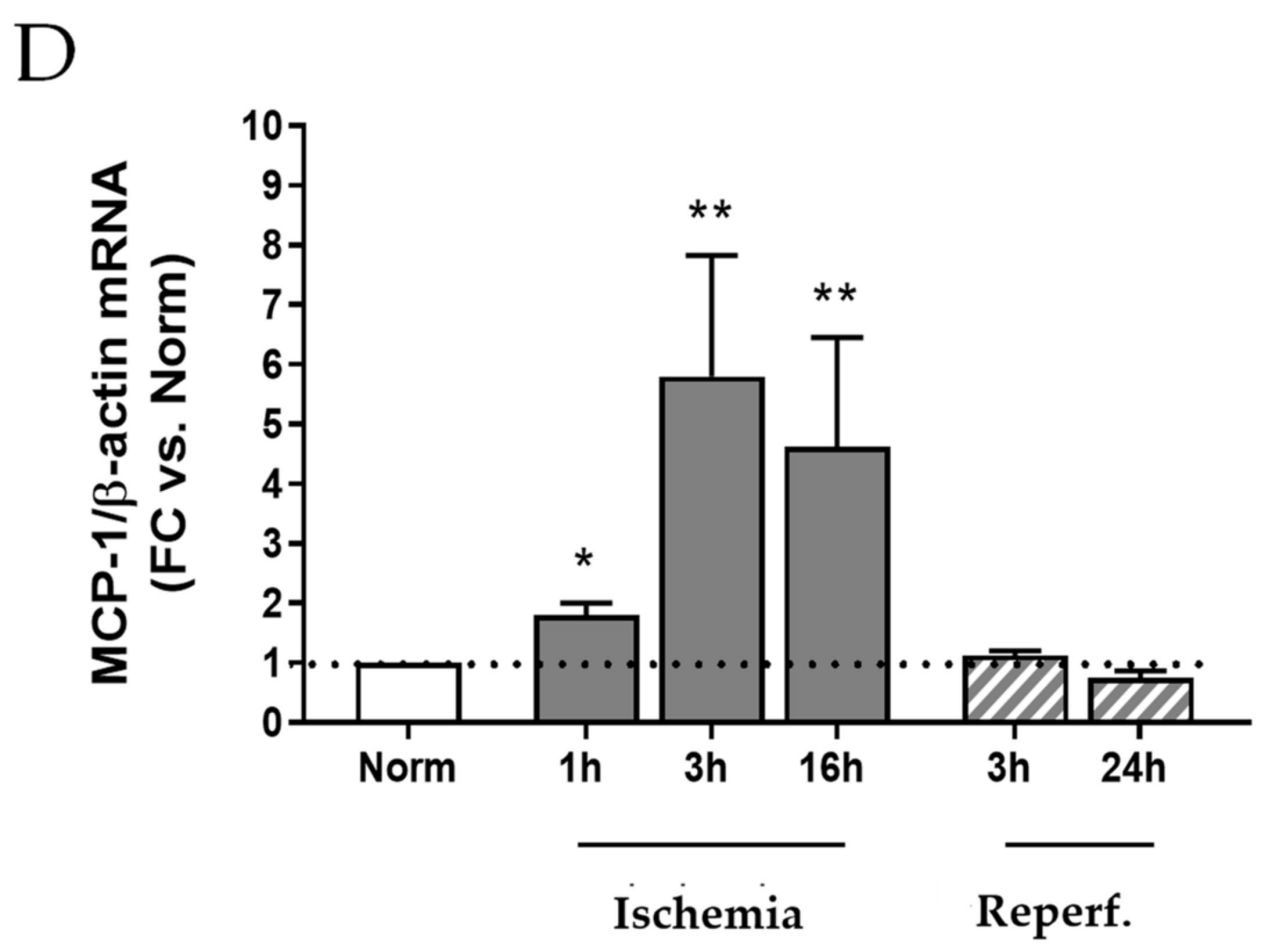
Mitochondrial function during I/R in HK-2 tubular renal cells. (**A**) Mitochondrial membrane potential and (**B**) mitochondrial mass in cells that underwent ischemia and reperfusion, analyzed by flow cytometry with the JC-1 and NAO probes, respectively; values are expressed as fold change (FC) versus normoxia at each time point; * *p* < 0.05, ** *p* < 0.01. (**C**) Immunofluorescence for PGC-1α, green signal (Alexa 488); magnification 20×. (**D**) MCP-1 mRNA levels after ischemia (1, 3, and 16 h) and reperfusion (3 and 24 h) by quantitative real-time PCR. The results have been normalized by expressing the number of transcript copies as a ratio to β-actin and indicated as fold changes (FC) in comparison to cells maintained in standard conditions for the same time (Normoxia); * *p* < 0.05, ** *p* < 0.01.

We then focused on mitochondrial balance, an early playmaker in cell response during I/R. Mitochondrial membrane potential, expressed as the ratio of the red/green fluorescence intensity of the JC-1 dye, was increased right after 20 h of ischemia and 24 h after reperfusion (35% and 48% increase versus normoxic control cells, *p* < 0.05 and *p* < 0.01, respectively) (Figure 5A). The nonyl acridine orange (NAO) signal, which indicates mitochondrial mass, was decreased by 20 h ischemia and even more deeply after 3 h of reperfusion (20% and 30%, *p* < 0.05 and *p* < 0.01, respectively); it recovered after 24 h of reperfusion (Figure 5B).

Consistently, the immunofluorescence signal for PGC-1α, a transcriptional coactivator involved in mitochondrial biogenesis, was decreased 20 h after ischemia and sensibly increased after 24 h of reflow-like conditions (Figure 5C). During ischemia, MCP-1 mRNA was significantly increased (*p* < 0.05 and *p* < 0.01) (up to five fold after 3 h) (Figure 5D).

### 2.6. Effects of I/R on Mstn and PCSK9 Expression in HK-2 Renal Tubular Cells

In rat kidneys, I/R induced Mstn accumulation in tubuli. Thus, we defined in vitro the dynamics of the Mstn gene and protein expression in tubular renal HK-2 cells challenged with I/R-like conditions in respect to control cells kept in standard culture conditions (“Normoxia”).

**Figure 6:**
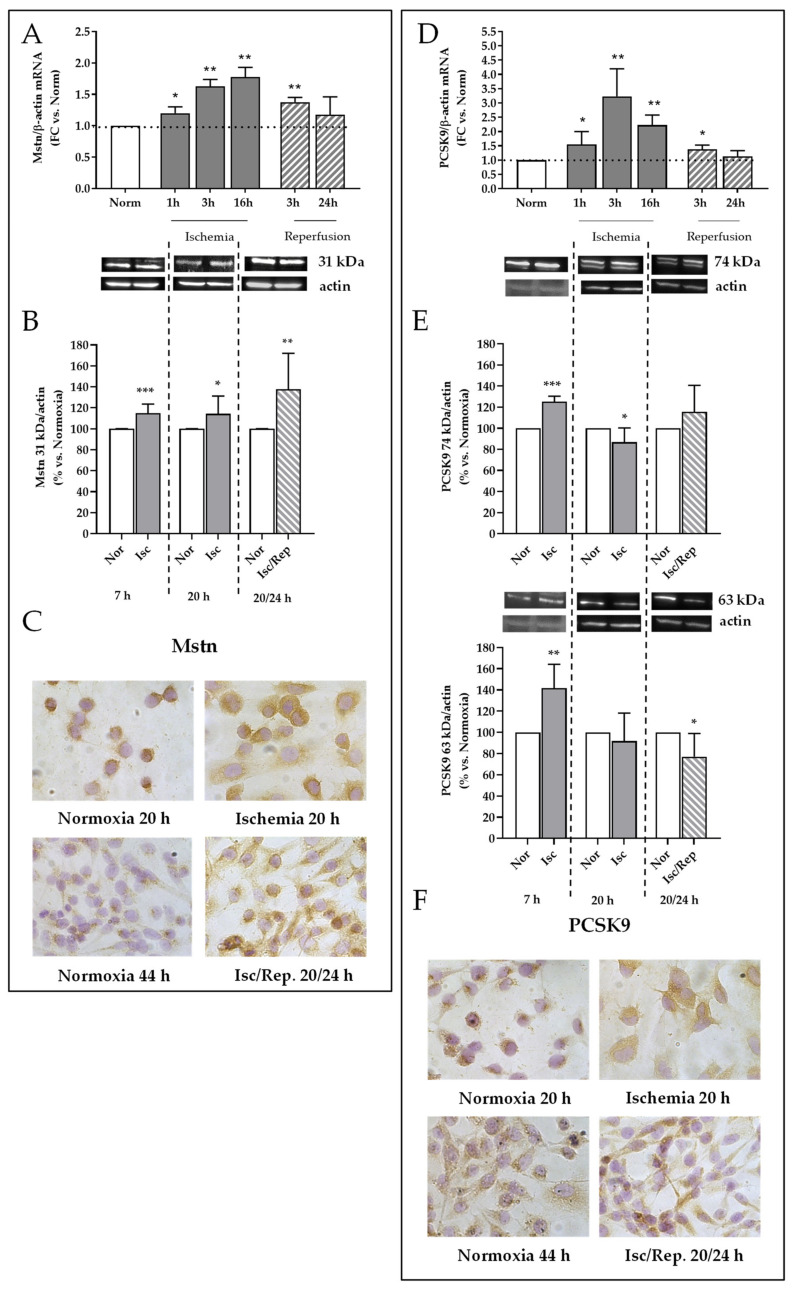
Mstn and PCSK9 during I/R in HK-2 tubular renal cells. Mstn: (**A**) mRNA levels after ischemia (1, 3, and 16 h) and reperfusion (3 and 24 h) by quantitative real-time PCR. The results have been normalized by expressing the number of transcript copies as a ratio to β-actin and indicated as fold changes (FC) in comparison to control cells (Normoxia); * *p* < 0.05, ** *p* < 0.01. (**B**) Western blot analysis of Mstn 31 kDa. Bands of interest were normalized against those obtained by membrane rehybridization with anti-total actin antibody. Representative immunoblots are shown. Data obtained from at least 3 independent experiments are expressed as % ± SEM in respect to control cells (Normoxia); * *p* < 0.05, ** *p* < 0.01, *** *p* < 0.001. (**C**) Immunocytochemistry for Mstn using DAB as chromogen; magnification 40×. PCSK9: (**D**) mRNA levels after ischemia (1, 3, and 16 h) and reperfusion (3 and 24 h) by quantitative real-time PCR. The results have been normalized by expressing the number of transcript copies as a ratio to β-actin and indicated as fold changes (FC) in respect to control cells (Normoxia); * *p* < 0.05, ** *p* < 0.01. (**E**) Western blot analysis of PCSK9 74 and 63 kDa isoforms. Bands of interest were normalized against those obtained by membrane rehybridization with anti-total actin antibody. Representative immunoblots are shown. Data obtained from at least 3 independent experiments are expressed as % ± SEM in respect to control cells (Normoxia); * *p* < 0.05, ** *p* < 0.01, *** *p* < 0.001. (**F**) Immunocytochemistry for PCSK9 using DAB as chromogen; magnification 40×.

Mstn mRNA was upregulated after 1 h of ischemia (1.3 fold changes, *p* < 0.05), and even more enhanced after 3 and 16 h (1.7 and 1.8 fold changes, *p* < 0.01); then, after 3 h reperfusion, mRNA levels were slightly diminished, and within 24 h, no difference was noticeable in comparison to control cells (Figure 6A). Western blot analysis revealed a different Mstn expression over time. Mstn was increased in I/R cells at each time point (7 and 20 h of ischemia and 24 h after reperfusion, *p* < 0.001, *p* < 0.05, and *p* < 0.01, respectively) (Figure 6B). Immunocytochemistry better highlighted the changes in cellular distribution: Mstn signal was enhanced in the cytoplasm after 20 h of ischemia in comparison to control cells, wherein immunopositivity was lower and mainly localized at the peri-nuclear level. After 24 h of reperfusion, Mstn positivity was still higher in I/R-treated cells (Figure 6C).

In our cell model, PCSK9 gene expression was significantly enhanced after 1, 3, and 16 h of ischemia (1.5, 3.5, and 2.4 fold changes, *p* < 0.05 and *p* < 0.01, respectively) Then, PCSK9 mRNA was diminished in reperfusion-like conditions, being still upregulated after 3 h (1.4 fold changes versus normoxia, *p* < 0.05) and no different from control cells after 24 h (Figure 6D). By western blot analysis, the immature precursor of PCSK9 is detected at 74 kDa, while the mature protein that underwent autocatalytic cleavage at 63 kDa. Seven hours of ischemia-like conditions increased both isoforms of PCSK9 (25% and 40% increase, versus normoxia, *p* < 0.001 and *p* < 0.01, respectively); after 20 h, the 74 kDa isoform displayed a 20% reduction (*p* < 0.05), while after 24 h of reflow-like conditions, only the mature protein (63 kDa) was reduced in comparison to normoxia control cells (*p* < 0.05) (Figure 6E). Immunocytochemistry evidenced the PCSK9 accumulation in the cytoplasm within 20 h of ischemia, when compared to control cells; after 24 h of incubation in reperfusion-like conditions, PCSK9 positivity was reduced in respect to the previous time point (hypoxia 20 h) and to normoxic control cells (Figure 6F).

### 2.7. Mstn Induces PCSK9 Expression in HK-2 Renal Tubular Cells

**Figure 7:**
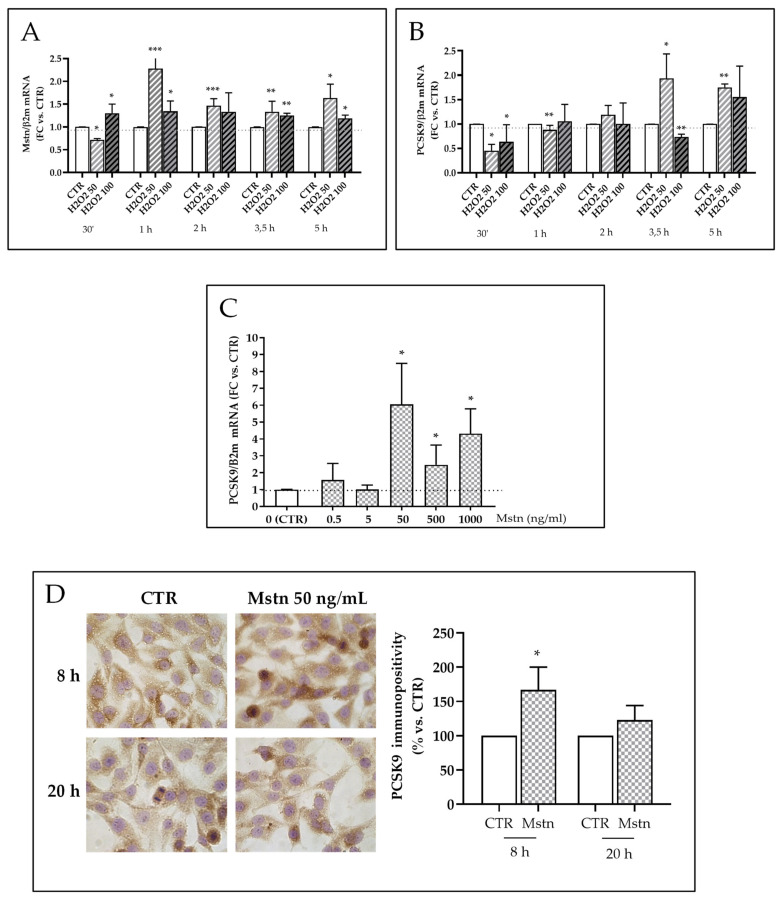
Mstn and PCSK9 in HK-2 tubular renal cells. (**A**) Mstn and (**B**) PCSK9 mRNA levels upon H_2_O_2_ treatment; * *p* < 0.05, ** *p* < 0.01, *** *p* < 0.001. (**C**) PCSK9 mRNA levels after a 5 h treatment with Mstn (0,5, 5, 50, 500, 1000 ng/mL). The results were obtained by quantitative real-time PCR, normalized expressing the number of transcript copies as a ratio to β2-microglobulin (β2-m) and indicated as fold changes (FC) in comparison to untreated control cells; * *p* < 0.05. (**D**) PCSK9 immunocytochemistry using DAB as chromogen, representative images (left side); immunostaining quantification, indicated as % of immunopositivity in respect to control cells (right side). Data are obtained from 3 independent experiments and the signal measured in 50 cells/samples at least; * *p* < 0.05.

Mstn is a known oxidative stress-associated factor. We first tested in our cell model (HK-2 cells) the timing of ROS production, Mstn, and PCSK9 mRNA expression upon H_2_O_2_ treatment. H_2_O_2_ 50 μM increased cell ROS within 30 min, while no significant differences were observed for longer incubation time or with H_2_O_2_ 100 μM (Supplementary Figure S3). Mstn was early upregulated within 30 min when treated with H_2_O_2_ 100 μM; after 1 h, its transcription reached the highest levels upon incubation with H_2_O_2_ 50 μM and remained consistently upregulated up to 5 h at both H_2_O_2_ concentrations (*p* < 0.05, *p* < 0.01, and *p* < 0.001) (Figure 7A). PCSK9 mRNA was diminished within the first hour of incubation and reached levels significantly higher than untreated control cells after 3.5 h of incubation with H_2_O_2_ 50 μM (*p* < 0.05 and *p* < 0.01) (Figure 7B).

We therefore investigated whether Mstn is able to induce PCSK9 expression in HK-2 cells. Five hours treatment with Mstn (50, 500, 1000 ng/mL) significantly increased PCSK9 mRNA levels (*p* < 0.05) (Figure 7C). Then, cells were treated with Mstn 50 ng/mL, corresponding to the median and most efficient concentration among those tested, and immunostained for PCSK9: positivity increased of 60% after a 8 h treatment (*p* < 0.05); then after a 20 h incubation, it decreased, and no significant difference between Mstn-treated and control cells was recorded (Figure 7D).

### 2.8. Effects of Mstn Silencing on PCSK9 Expression Induced by Ischemia in HK-2 Renal Tubular Cells

**Figure 8:**
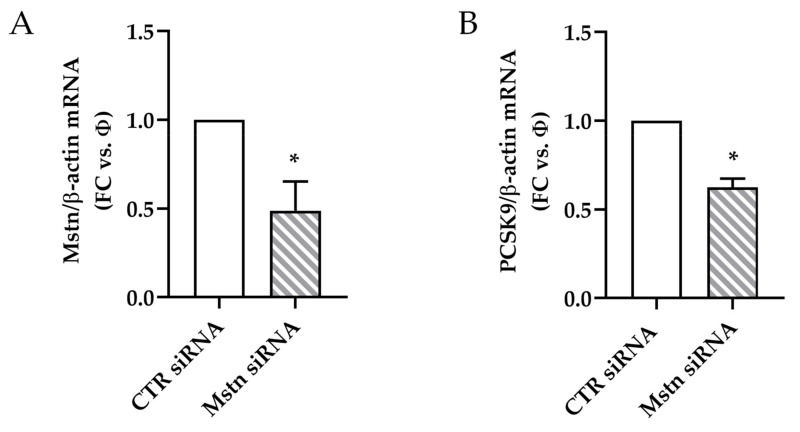

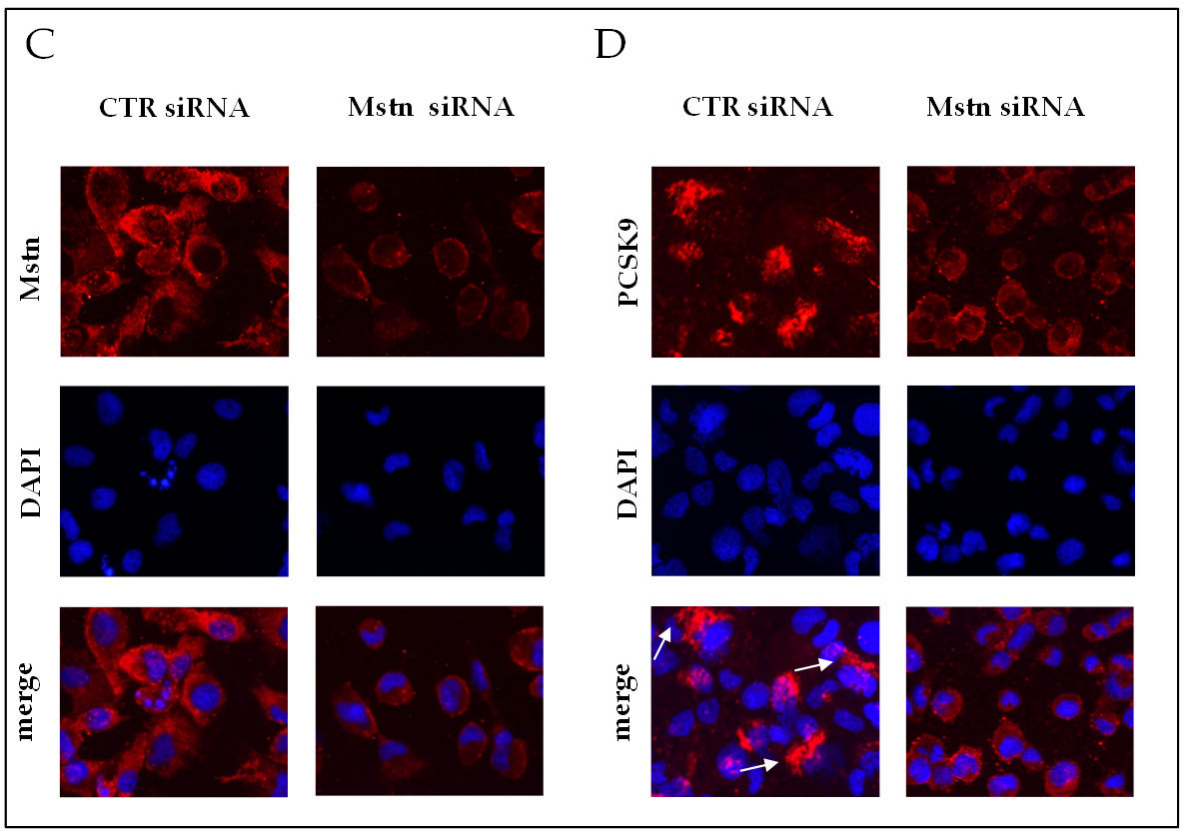
PCSK9 expression during I/R in Mstn-silenced HK-2 tubular renal cells. (**A**) Mstn and (**B**) PCSK9 mRNA levels in control siRNA cells (CTR siRNA) and Mstn siRNA cells after 3 h of ischemia. The results are obtained by quantitative real-time PCR, normalized expressing the number of transcript copies as a ratio to β-actin and indicated as fold changes (FC) in respect to CTR siRNA cells (Φ); * *p* < 0.05. (**C**) Mstn and (**D**) PCSK9 immunofluorescence (red signal, Alexa fluor 555) in control siRNA cells (CTR siRNA) and Mstn siRNA cells after 6 h of ischemia. White arrows indicate the cortical distribution of PCSK9 aggregates in CTR siRNA cells; magnification 40×.

Finally, we tested whether Mstn silencing by transfecting HK-2 cells with Mstn siRNA (Mstn siRNA cells) may result in a reduction of ischemia-induced PCSK9; after 3 h, the Mstn mRNA was significantly reduced (0.45 folds in comparison to siRNA control cells, *p* < 0.05) (Figure 8A). Consistently with our hypothesis, PCSK9 mRNA was also significantly reduced (0.55 fold in comparison to siRNA control cells, *p* < 0.05) (Figure 8B).

After 6 h of ischemia, the immunofluorescence was reduced for both Mstn and PCSK9 in Mstn-silenced cells, according to the gene expression. The signals were differently distributed: in control siRNA cells, Mstn accumulated in the cytoplasm and PCSK9 formed large aggregates with cortical localization in some cells. In Mstn siRNA cells, both Mstn and PCSK9 fluorescence intensity were reduced, homogeneously distributed along the perinuclear compartment (Figure 8C,D).

MCP-1 mRNA, although modulated by ischemia, was unaffected by Mstn silencing (Supplementary Figure S4).

## 3. Discussion

Surgical repair of supra/juxtarenal abdominal aortic aneurysms (AAAs) is associated with high risk of post-operative renal failure, becoming dysfunction in 8 to 41% and dialysis in 4 to 7% of cases, rates that constitute a critical clinical issue [16,17,18,19,20,21].

Beside preoperative risk factors (age, widespread atherosclerosis, the use of nephrotoxic therapeutic drugs, and iodinated contrast agents for imaging), a leading cause of renal failure is tubular necrosis due to the cross-clamping of the aorta above or close to the renal arteries [22].

An underlying mechanism of this process involves ischemia/reperfusion (I/R), a two-step phenomenon that primarily affects the mitochondria; in this context, they undergo major changes, loss of energy, the derangement of the ionic homeostasis, and the production of reactive oxygen species (ROS) that may ultimate lead to cell death [1].

Many preclinical studies identified redox imbalance and oxidative damage as the main driver of I/R injury; however, their comparison with clinical findings displayed some discrepancies, which make the antioxidant agents challenged for protective purposes of controversial usefulness [23]. As demonstrated by de Vries and colleagues [7], these different results may be explained by a higher redox buffer capacity of humans in comparison to animal models [24] and by the presence of other mechanisms that, together with ROS overproduction, may be dysregulated during I/R [25,26].

The different pathways responsible for the tubular damage are not fully elucidated yet and their comprehension would suggest strategies of protection to minimize the post-operative effects of renal I/R.

With this perspective, we investigated the role of myostatin (Mstn) and proprotein convertase subtilisin/kexin type 9 (PCSK9), mediators involved in the dysregulation of metabolism and catabolic/inflammatory disorders, so far unexplored in the context of renal I/R. We first evaluated in vivo whether Mstn and PCSK9 were affected in a rat model of I/R; then, to better address our observation, we evaluated in vitro the timing of Mstn and PCSK9 gene and protein expression and the mitochondrial functions in HK-2 cells exposed to I/R-like conditions.

In vivo, 4 h after reflow, kidneys that underwent I/R had higher Mstn and PCSK9 protein levels; one day later, they displayed focal tubular necrosis, the enhanced expression of N-tyrosine, a marker of ROS accumulation and the nuclear relocation of peroxisome proliferator-activated receptor gamma coactivator-1alpha, indicating the occurrence of oxidative stress and an attempt to renew the mitochondrial pool in tubular cells, respectively. Mstn mRNA was downregulated, and immunopositivity increased mainly in tubular cells. As opposed to Mstn transcription and protein expression, PCSK9 mRNA was increased and the immune signal reduced. Systemically, plasma levels of PCSK9 were significantly higher. Taken together, these events let us hypothesize a mechanism of transcription/secretion/release of the PCSK9 protein.

Finally, kidneys from I/R rats had an increased monocyte chemoattractant protein-1 (MCP-1) transcription. In vitro, both ischemia and reperfusion induced ROS overproduction and transiently affected mitochondria, increasing their membrane potential and reducing their mass. The basal mitochondrial pool and function were restored within 24 h of perfusion-like conditions.

The increase of mitochondrial membrane potential has been reported also in the tubular cells of young spontaneous hypertensive compared with normotensive rats [27], indicating this as an altered bioenergetic profile also operative early in hypertensive renal damage. In agreement with the decrease of mitochondrial mass during ischemia and short after reperfusion, cell cycle arrest in G0/G1 was observed after ischemia, accounting for an autophagy (mitophagy) process.

In HK-2 cells, Mstn and PCSK9 gene expression was upregulated early after exposure to ischemia-like condition, to restore their basal level during reperfusion. Mstn is both sensitive to and inducer of free radical compounds and mitochondrial metabolic alteration [28,29].

Consistent with the histological findings on rat kidneys, an increase of Mstn content and the depletion of PCSK9 after I/R was observed in HK-2 cells. When incubated with H_2_O_2_, a prooxidant stressor, they overexpress Mstn first and, later on, PCSK9 mRNA. Incubation with recombinant Mstn enhanced PCSK9 mRNA and protein content; over time, immunostaining slightly decreased, suggesting also in vitro a mechanism of production and release.

As proof of concept of a Mstn-dependent expression of PCSK9, we observed reduced levels of PCSK9 in Mstn-silenced (Mstn siRNA) cells exposed to ischemia when compared to control cells (CTR siRNA). By contrast, MCP-1 mRNA was unaffected by Mstn silencing. This observation indicates that, although multiple metabolic and inflammatory pathways participate to I/R injury, tackling Mstn allows a targeted modulation only of some of them, in our case PCSK9.

Taken together, our results demonstrate that Mstn and PCSK9 are early induced during surgical ischemia in rat kidneys and in HK-2 tubular renal cells exposed to I/R-like conditions and associated to mitochondrial impairment.

Previous studies already proved separately the benefit of Mstn and PCSK9 modulation in different clinical settings; in sarcopenia, the silencing of Mstn has been shown to restrain the proteasomal-mediated catabolism of intracellular proteins, the muscle wasting, and to potentiate basal antioxidant enzyme levels [30]. Basally expressed in heart, Mstn signal is upregulated after myocardial infarction, through the canonical (SMAD proteins-dependent) and noncanonical pathway; in turn, it upregulates atrophy-related atrogenes or autophagy genes, resulting in proteasome-dependent muscle protein degradation. When utilizing Mstn-deficient mice, or a decoy for its receptor ACVR2B-Fc, the absence of Mstn signal improves cardiac function after myocardial infarction [31,32].

PCSK9 is significantly upregulated in I/R-exposed murine cardiomyocytes [9,33] and in the border zone of infarcted myocardium one week after left coronary artery ligation [9]. The release of PCSK9 during cardiac ischemia results in cell death and dysfunction; inflammatory stimuli (i.e., IL-1β) are considered to be powerful inducers for PCSK9 secretion in both macrophages and tissues such as heart and aorta [9,34]. Interestingly, PCSK9 itself might be an important inflammatory mediator by inducing intracellular cholesterol accumulation in macrophages and other immune cell types, increasing toll-like receptor function and amplifying inflammatory reactions that eventually lead to the progression of coronary atherosclerosis [35].

Clinical trials demonstrated the benefit of PCSK9 inhibition: administration of the anti-PCSK9 antibody evolocumab to patients with a recent myocardial infarction significantly reduced the risk of the composite outcome of cardiovascular death [36]; the combination of a PCSK9 antibody (alirocumab) with statin therapy reduced the occurrence of both type 1 and type 2 myocardial infarction in patients with recent acute coronary syndrome and dyslipidemia [37].

In the kidney, PCSK9 is constitutively expressed during nephrogenesis; when administered exogenously, it reduces tissue low-density lipoprotein receptor expression. Its function, however, remains largely unknown. The clinical consequences of renal PCSK9 fluctuation so far have not been assessed, mainly because of multiple interconnection and indirect feedback with a plethora of factors involved in inflammatory and metabolic disorders [38].

To our knowledge, so far the link between Mstn and PCSK9 expression in renal I/R remained unexplored; our work is the first to provide mechanistic evidence of their crosstalk during I/R, suggesting them as new targets for protective strategies against post-surgical complications.

Although promising, our findings need to be further investigated. The main limitations are due to the lack of information on long term effects of Mstn and PCSK9 induction and/or modulation during recovery from I/R in vivo; looking at the in vitro model, although in our setting it paralleled the main results obtained in vivo, it does not allow us to evaluate the contribution of the extracellular matrix and in vivo moiety in modulating the signal of Mstn and PCSK9 [39].

Summing up, our work provides hints on the role of two emerging mediators of cardiovascular damage, Mstn and PCSK9; found early upregulated in animal and cellular model of renal I/R, their induction and effects may be restrained with different approaches. Future studies evaluating their targeting, alone or in combination, may provide a better comprehension of the Mstn and PCSK9 in the pathogenesis of renal I/R and indicate novel protective approaches against post-operative and late clinical complications after aortic cross clamping.

## 4. Materials and Methods

### 4.1. Animal Model

Male Wistar rats (home-bred from stock originally obtained from Harlan Laboratories, San Pietro al Natisone, Italy), 8–10 weeks old, were housed in a pathogen-free environment at Animal Facility, IRCCS Ospedale Policlinico San Martino, Genoa. Water and regular diet were available ad libitum. Four separate groups were used to acquire data: the ischemia/reperfusion group (I/R 30 min/4 h, *n* = 3) with the relative control group (sham, *n* = 3) and the ischemia/reperfusion group (I/R 30 min/24 h, *n* = 4) with the relative control group (sham, *n* = 3).

The surgical induction of I/R injury was performed as follows: animals were anesthetized with diazepam (subcutaneous—5 mg/kg), ketamine (intraperitoneal—75 mg/kg), and xylazine (intraperitoneal—10 mg/kg), submitted to a surgical procedure of supraceliac aorta clamping for 30 min to induce complete abdominal ischemia, as previously described [8]. As control, animals of the sham-operated groups underwent the surgical procedure for aorta isolation, without aortic clamping and blood flow interruption. After 30 min of ischemia, the clamp was removed and the blood flow restored for 4 or 24 h. During ischemia and reperfusion, animals were maintained in their own cages, under anesthesia and warm light for body temperature maintenance. Finally, animals were euthanized by pentobarbital sodium overdose (intravenous injection—150 mg/kg) for organ collection.

Immediately after explantation, kidneys were dissected and snap-frozen in dry ice until storage at −80 °C for protein and RNA extraction or kept in cold phosphate buffered saline (PBS) for 40 min, changing the buffer solution 3 times, and then fixed in cold paraformaldehyde for paraffin embedding. Rat kidney homogenization was obtained with a TissueLyser (Qiagen Sciences, Germantown, MD, USA). The study was conducted according to the Guide for the Care and the Use of Laboratory Animals, published by the US National Institutes of Health (NIH Publication No. 85-23, updated in 2011). Furthermore, the animal experimental protocol was in accordance with ARRIVE guidelines and approved by the Italian Ministry of Health (75/2018-PR: #22418.76).

### 4.2. Histopathological Examination

Standard histopathological techniques were followed for processing the fixed kidney tissue and the preparation of paraffin blocks. The paraffin sections were dewaxed, hydrated, and stained with hematoxylin and eosin or periodic acid-Schiff (PAS) and counterstained with hematoxylin to detect tissue damage and tubular necrosis. Specimens were examined in a blinded manner by two pathologists independently under light microscopy. Briefly, at least 10 fields/section (20× and 40× magnification) were checked, as described by Speir and colleagues [40].

### 4.3. Immunohistochemistry

Paraffin sections (5 μm) of 2% paraformaldehyde-fixed tissue were analyzed for nitrotyrosine (N-tyr) and peroxisome proliferator-activated receptor gamma coactivator-1alpha (PGC-1α) mouse monoclonal antibodies, at the dilution of 1:100 (Santa Cruz Biotechnology, Inc., Dallas, TX, USA), Myostatin (Mstn) and proprotein convertase subtilisin/kexin type 9 (PCSK9) rabbit polyclonal antibodies, at the dilution of 1:80 and 1:100, respectively (both from Proteintech, Manchester, UK). Immunostainings were completed with the appropriate secondary antibody using the streptavidin-peroxidase method and the DAB (3,3′-diaminobenzidine) chromogen, as previously described [41].

### 4.4. PCSK9 and Creatinine Quantification in Plasma

PCSK9 levels in plasma were measured by a rat ELISA kit (# MBS1600428, MyBioSource, Inc., San Diego, CA, USA), whereas creatinine levels were quantified by Cr assay kit (#EU3134, Wuhan Fine Biotech Co., Ltd., Wuhan, China) according to the manufacturer’s protocol.

### 4.5. Cell Cultures and Treatments

Cell cultures: proximal tubular epithelial cells from human kidney (HK-2, ATCC CRL-2190) were obtained from ATCC (Manassas, VA, USA). Cells were grown in complete medium (Dulbecco’s Modified Eagle Medium/Nutrient Mixture F-12 Ham, DMEM/F-12, supplemented with 5% [*v*/*v*] fetal bovine serum, FBS, 100 U/mL penicillin-streptomycin, 2 mmol L-glutamine, 5 μg/mL insulin, 5 μg/mL transferrin, 5 ng/mL sodium selenite, 5 pg/mL T3, 5 ng/mL hydrocortisone, 5 pg/mL PGE1, and 10 ng/mL epidermal growth factor) at 37 °C in a humidified 5% CO_2_ chamber.

Cell treatments:

- ischemia-like conditions were reproduced by replacing complete medium with DMEM/F12 alone and incubating cells in a modular incubator chamber (Billups-Rothenberg, CA, USA), filled with an anoxic gas mixture (95% N_2_ and 5% CO_2_), at 37 °C for at most 20 h;
- reperfusion-like conditions were reproduced by exposing cells to fresh complete medium in standard conditions (atmospheric air supplemented with 5% CO_2_) up to 24 h;
- H_2_O_2_-dependent effects on Mstn and PCSK9 expression were evaluated by treating cells with two different concentrations of H_2_O_2_ (50 and 100 μM) (Carlo Erba, Milan, Italy) for different times (30 min, 1, 2, 3,5, and 5 h) in standard culture conditions;
- Mstn-dependent effects on PCSK9 expression were evaluated by adding recombinant Mstn (0.5–1000 ng/mL) (Peprotech, LiStarFish, Cernusco sul Naviglio, Italy) to complete culture medium for 5, 8, and 20 h in standard culture conditions;
- Mstn silencing was obtained by the transfection of HK-2 cells with 30 nM Mstn specific siRNA (Mstn siRNA) or negative control siRNA (CTR siRNA), also named “Φ”, using lipofectamine (Thermo Fisher Scientific, Waltham, MA, USA), according to the manufacturer’s protocol. Cells were then incubated at 37 °C in a CO_2_ incubator for 24 h until they were ready for assay. The efficacy of knockdown was determined by real-time PCR and immunofluorescence.

### 4.6. Flow Cytometry

Nuclear DNA content: cells were permeabilized, and nuclear DNA content was stained with a hypotonic solution of propidium iodide (50 μg/mL in sodium citrate/Triton X-100) for 1 h at room temperature. Cell cycle analysis was performed using the ModFit LT 4.0 software (Verity Software House, Topsham, ME, USA).

ROS production: the fluorogenic probe CellROX™ Deep Red Reagent (Thermo Fisher Scientific, Waltham, MA, USA), which reacts with ROS, was used to detect the oxidative stress in HK-2 cells. Cells were stained according to the manufacturer’s protocol and measured.

Mitochondrial membrane potential: changes in mitochondrial membrane potential were assayed using 5,5,6,6-tetrachloro-1,1,3,3-tetraethylbenzimidazolylcarbocyanine iodide (JC-1) dye (Thermo Fisher Scientific, Waltham, MA, USA), which selectively enters the mitochondria. Upon excitation at 480 nm, JC-1 displays green fluorescence in the monomeric form (at low membrane potential), while at high-membrane potential the probe forms J-aggregates with red fluorescence. Living cells were detached with trypsin, stained, and analyzed, as previously described [42].

Mitochondrial mass: as a control of mitochondrial mass, cells were incubated with 10 µM nonyl acridine orange (NAO) (Sigma-Aldrich, Saint Louis, MO, USA) for 10 min in the dark at room temperature, washed twice in PBS, and immediately analyzed.

FACS analysis was performed on 0.5–1 × 10^6^ cells/mL per sample with an Attune Acoustic Focusing Cytometer (Thermo Fisher Scientific, Waltham, MA, USA).

### 4.7. Immunocytochemistry

HK-2 cells grown on chamber slides to subconfluence were exposed to different stimuli, as described above. After a two minute fixation in cold methanol, cells were incubated with anti-Mstn GDF8/myostatin polyclonal antibody (dilution 1:100) or anti-PCSK9 antibody (dilution 1:250), both from Proteintech (Manchester, UK). Slides were counterstained with hematoxylin and examined by light microscopy.

### 4.8. Immunofluorescence

After exposure to different stimuli, HK-2 grown on chamber slides were fixed in cold methanol and then incubated with anti-N-tyr and anti-PGC-1α mouse monoclonal antibodies (Santa Cruz Biotechnology, Inc., Dallas, TX, USA) at a dilution of 1:100 or with anti-Mstn (dilution 1:100) and anti-PCSK9 (dilution 1:160) rabbit polyclonal antibodies (Proteintech, Manchester, UK). As secondary antibodies, goat-anti-mouse Alexa Fluor 488 or goat-anti-rabbit Alexa Fluor 555 were used (Invitrogen, Carlsbad, CA, USA).

### 4.9. Image Analysis

In immunohistochemical/immunocytochemical/immunofluorescence staining, the positivity was evaluated by image analysis performed using the Leica Q500 MC Image Analysis System (Leica, Cambridge, UK), as previously described [13].

### 4.10. mRNA Analysis

The total RNA was extracted using the QIAzol Lysis Reagent (Qiagen Sciences, Germantown, MD, USA) and the concentration and integrity of each sample were evaluated on a NanoDrop ND-1000 Spectrophotometer (NanoDrop Technologies Inc., Wilmington, DE, USA). A total of 1 μg RNA was used for cDNA synthesis.

### 4.11. cDNA Reverse Transcription and Quantitative Real-Time PCR

cDNA synthesis was performed using the iScript cDNA synthesis kit RT (Bio-Rad Laboratories Inc., Hercules, CA, USA). Primers were obtained from Tib Molbiol S.r.l. (Genoa, Italy) and sequences are reported in Table 1. PCR amplification was carried out in a total volume of 10 μL, containing 1 μL of cDNA solution, 5 μL of SYBR Master Mix solution (Eppendorf, Hamburg, Germany), 0.25 μM of each primer, and nuclease-free water. Based on their stability in respect to treatments, β-actin (for exposure to ischemia) or β2-microglobulin (for exposure to reperfusion) were quantified and used to normalize the expression of the other genes. The ∆∆CT method of relative quantification was used to determine the fold change in expression. Assays were run in triplicate using a Universal PCR Master Mix on a MasterCycler RealPlex PCR system (Eppendorf, Hamburg, Germany).

### 4.12. Western Blotting

Equal amounts of proteins, estimated by Quantum Protein (Euroclone, Pero, Italy), from rat kidney homogenates or HK-2 cell lysates were run on Novex WedgeWell 8–16% Tris-Glycine gels (Thermo Fisher Scientific, Waltham, MA, USA). Then, samples were electrotransferred to PVDF membranes (Bio-Rad Laboratories Inc., Hercules, CA, USA) and saturated with 5% [*m*/*v*] non-fat milk (Euroclone, Pero, Italy) in TPBS (phosphate buffered saline with 0.005% [*v*/*v*] Tween^®^20) for 1 h at room temperature. The membranes were incubated with anti-Mstn GDF8/myostatin polyclonal antibody (dilution 1:1000) or anti-PCSK9 antibody (dilution 1:500), both from Proteintech (Manchester, UK) for 16 h at 4 °C. The membranes were then incubated with an anti-rabbit IgG, HRP-linked antibody for 1 h at room temperature (Cell Signaling Technology, Danvers, MA, USA) (dilution 1:2000). The bands were detected by Clarity Western ECL Substrate (Bio-Rad Laboratories Inc., Hercules, CA, USA) by using a chemiluminescence imager (Alliance Q9, UVITEC, Cambridge, UK). Total actin was used as reference protein and was detected by using an anti-actin antibody (Santa Cruz Biotechnology, Inc., Dallas, TX, USA) (dilution 1:500) and a donkey anti-goat IgG-HRP (Santa Cruz Biotechnology, Inc., Dallas, TX, USA) as secondary antibody (dilution 1:7000). Protein bands were quantified by densitometry using the Alliance system (Uvitec, Cambridge, UK).

### 4.13. Statistics

In vitro experiments were performed at least 3 times. Summary data are expressed as mean ± standard deviation and compared by Student’s *t*-test. Statistical significance was set at *p* < 0.05. All statistical analyses were performed using GraphPad Prism version 5.00 for Windows (GraphPad Software, San Diego, CA, USA).

## Figures and Tables

**Table 1:** Primer sequences.

Name	Species	Accession number	Forward	Reverse
Proprotein convertase subtilisin/kexin type 9 (PCSK9)	rat	NM_199253	5′ TCT.AAG AGA TAC ACC TCC ACC TG 3′	5′ ACT CAT TAG TCT TCG CCC AGA 3′
Myostatin (Mstn)	rat	NM_019151.1	5′GGCACTGGTATTTGGCAGAGT 3′	5′AGGGATTCAGCCCATCTTCTC 3′
Monocyte chemoattractant protein-1 (MCP-1)	rat	NM_031530.1	5′TGACAAATACTACAGCTTCTTTGGG 3′	5′CAGTTAATGCCCCACTCACCT 3′
β2-microglobulin (β2-m)	rat	NM_012512	5′ TAG CAG TTG AGG AAG TTG GG 3′	5′ TGA TCT TTC TGG TGC TTG TCT C 3′
Proprotein convertase subtilisin/kexin type 9 (PCSK-9)	human	NM_174936	5′ AGG GGA GGA CAT CAT TGG TG 3′	5′-CAG GTT GGG GGT CAG TAC C 3′
Myostatin (Mstn)	human	NM_005259.	5′CCAAAGCTCCTCCACTCCG 3′	5′GGGAGTACAGCAAGGGCC 3′
Monocyte chemoattractant protein-1 (MCP-1)	human	NM_002982	5′ACCGAGAGGCTGAGACTAAC 3′	5′AATGAAGGTGGCTGCTATGAG 3′
β2-microglobulin (β2-m)	human	NM_004048	5′ CCA GCG TAC TCC AAA GAT TCA 3′	5′ TGC TCC ACT TTT TCA ATT CTC TC 3′
β-actin	human	NM_001101.2	5′CCTCGCCTTTGCCGATCC 3′	5′CTCGTCGCCCACATAGGAAT 3′

## Supplementary Materials

The following are available online at https://www.mdpi.com/article/10.3390/ijms22189884/s1.

## References

[1] Jassem W., Heaton N.D. (2004). The role of mitochondria in ischemia/reperfusion injury in organ transplantation. Kidney Int..

[2] Zhao Z., Tang Z., Zhang W., Liu J., Li B. (2017). Magnesium isoglycyrrhizinate protects against renal-ischemia-reperfusion injury in a rat model via anti-inflammation, anti-oxidation and anti-apoptosis. Mol. Med. Rep..

[3] Liu X., Murphy M.P., Xing W., Wu H., Zhang R., Sun H. (2018). Mitochondria-targeted antioxidant MitoQ reduced renal damage caused by ischemia-reperfusion injury in rodent kidneys: Longitudinal observations of T_2_-weighted imaging and dynamic contrast-enhanced MRI. Magn. Reson. Med..

[4] Yamamoto S., Hagiwara S., Hidaka S., Shingu C., Goto K., Kashima K., Noguchi T. (2011). The antioxidant EPC-K1 attenuates renal ischemia-reperfusion injury in a rat model. Am. J. Nephrol..

[5] Zhang Y., Rong S., Feng Y., Zhao L., Hong J., Wang R., Yuan W. (2017). Simvastatin attenuates renal ischemia/reperfusion injury from oxidative stress via targeting Nrf2/HO-1 pathway. Exp. Ther. Med..

[6] Nezu M., Souma T., Yu L., Suzuki T., Saigusa D., Ito S., Suzuki N., Yamamoto M. (2017). Transcription factor Nrf2 hyperactivation in early-phase renal ischemia-reperfusion injury prevents tubular damage progression. Kidney Int..

[7] De Vries D.K., Kortekaas K.A., Tsikas D., Wijermars L.G., van Noorden C.J., Suchy M.T., Cobbaert C.M., Klautz R.J., Schaapherder A.F., Lindeman J.H. (2013). Oxidative damage in clinical ischemia/reperfusion injury: A reappraisal. Antioxid. Redox Signal..

[8] Pane B., Gazzola V., Spinella G., Bagnato P., Grillo F., Vellone V.G., Palombo D. (2018). Inflammatory response modulation through a PPARγ agonist during surgically induced visceral ischemia in an animal model. Ann. Vasc. Surg..

[9] Ding Z., Wang X., Liu S., Shahanawaz J., Theus S., Fan Y., Deng X., Zhou S., Mehta J.L. (2018). PCSK9 expression in the ischaemic heart and its relationship to infarct size, cardiac function, and development of autophagy. Cardiovasc. Res..

[10] Palee S., McSweeney C.M., Maneechote C., Moisescu D.M., Jaiwongkam T., Kerdphoo S., Chattipakorn S.C., Chattipakorn N. (2019). PCSK9 inhibitor improves cardiac function and reduces infarct size in rats with ischaemia/reperfusion injury: Benefits beyond lipid-lowering effects. J. Cell. Mol. Med..

[11] Pomiès P., Blaquière M., Maury J., Mercier J., Gouzi F., Hayot M. (2016). Involvement of the FoxO1/MuRF1/Atrogin-1 signaling pathway in the oxidative stress-induced atrophy of cultured chronic obstructive pulmonary disease myotubes. PLoS ONE.

[12] Castillero E., Akashi H., Wang C., Najjar M., Ji R., Kennel P.J., Sweeney H.L., Schulze P.C., George I. (2015). Cardiac myostatin upregulation occurs immediately after myocardial ischemia and is involved in skeletal muscle activation of atrophy. Biochem. Biophys. Res. Commun..

[13] Verzola D., Milanesi S., Bertolotto M., Garibaldi S., Villaggio B., Brunelli C., Balbi M., Ameri P., Montecucco F., Palombo D. (2017). Myostatin mediates abdominal aortic atherosclerosis progression by inducing vascular smooth muscle cell dysfunction and monocyte recruitment. Sci. Rep..

[14] Verzola D., Milanesi S., Viazzi F., Ansaldo F., Saio M., Garibaldi S., Carta A., Costigliolo F., Salvidio G., Barisione C. (2020). Enhanced myostatin expression and signalling promote tubulointerstitial inflammation in diabetic nephropathy. Sci. Rep..

[15] Shanley P.F., Rosen M.D., Brezis M., Silva P., Epstein F.H., Rosen S. (1986). Topography of focal proximal tubular necrosis after ischemia with reflow in the rat kidney. Am. J. Pathol..

[16] Knott A.W., Kalra M., Duncan A.A., Reed N.R., Bower T.C., Hoskin T.L., Oderich G.S., Gloviczki P. (2008). Open repair of juxtarenal aortic aneurysms (JAA) remains a safe option in the era of fenestrated endografts. J. Vasc. Surg..

[17] Jean-Claude J.M., Reilly L.M., Stoney R.J., Messina L.M. (1999). Pararenal aortic aneurysms: The future of open aortic aneurysm repair. J. Vasc. Surg..

[18] Sarac T.P., Clair D.G., Hertzer N.R., Greenberg R.K., Krajewski L.P., O’Hara P.J., Ouriel K. (2002). Contemporary results of juxtarenal aneurysm repair. J. Vasc. Surg..

[19] Jongkind V., Yeung K.K., Akkersdijk G.J.M., Heidsieck D., Reitsma J.B., Tangelder G.J., Wisselink W. (2010). Juxtarenal aortic aneurysm repair. J. Vasc. Surg..

[20] Bicknell C.D., Cowan A.R., Kerle M.I., Mansfield A.O., Cheshire N.J.W., Wolfe J.H.N. (2003). Renal dysfunction and prolonged visceral ischaemia increase mortality rate after suprarenal aneurysm repair. Br. J. Surg..

[21] Norwood M.G.A., Polimenovi N.M., Sutton A.J., Bown M.J., Sayers R.D. (2004). Abdominal aortic aneurysm repair in patients with chronic renal disease. Eur. J. Vasc. Endovasc. Surg..

[22] Wahlberg E., DiMuzio P.J., Stoney R.J. (2002). Aortic clamping during elective operations for infrarenal disease: The influence of clamping time on renal function. J. Vasc. Surg..

[23] Bartekova M., Barancik M., Ferenczyova K., Dhalla N.S. (2018). Beneficial effects of N-acetylcysteine and N-mercaptopropionylglycine on ischemia reperfusion injury in the heart. Curr. Med. Chem..

[24] Demetrius L. (2005). Of mice and men. When it comes to studying ageing and the means to slow it down, mice are not just small humans. EMBO Rep..

[25] Ralto K.M., Rhee E.P., Parikh S.M. (2020). NAD^+^ homeostasis in renal health and disease. Nat. Rev. Nephrol..

[26] Pefanis A., Ierino F.L., Murphy J.M., Cowan P.J. (2019). Regulated necrosis in kidney ischemia-reperfusion injury. Kidney Int..

[27] Lee H., Abe Y., Lee I., Shrivastav S., Crusan A.P., Hüttemann M., Hopfer U., Feld R.A. (2014). Increased mitochondrial activity in renal proximal tubule cells from young spontaneously hypertensive rats. Kidney Int..

[28] Enoki Y., Watanabe H., Arake R., Sugimoto R., Imafuku T., Tominaga Y., Ishima Y., Kotani S., Nakajima M., Tanaka M. (2016). Indoxyl sulfate potentiates skeletal muscle atrophy by inducing the oxidative stress-mediated expression of myostatin and atrogin-1. Sci. Rep..

[29] Liu Y., Cheng H., Zhou Y., Zhu Y., Bian R., Chen Y., Li C., Ma Q., Zheng Q., Zhang Y. (2013). Myostatin induces mitochondrial metabolic alteration and typical apoptosis in cancer cells. Cell Death Dis..

[30] Sriram S., Subramanian S., Sathiakumar D., Venkatesh R., Salerno M.S., McFarlane C.D., Kambadur R., Sharma M. (2011). Modulation of reactive oxygen species in skeletal muscle by myostatin is mediated through NF-κB. Aging Cell.

[31] Lim S., McMahon C.D., Matthews K.G., Devlin G.P., Elston M.S., Conaglen J.V. (2018). Absence of myostatin improves cardiac function following myocardial infarction. Heart Lung Circ..

[32] Castillero E., Akashi H., Najjar M., Ji R., Brandstetter L.M., Wang C., Liao X., Zhang X., Sperry A., Gailes M. (2020). Activin type II receptor ligand signaling inhibition after experimental ischemic heart failure attenuates cardiac remodeling and prevents fibrosis. Am. J. Physiol. Heart Circ. Physiol..

[33] Yang C.L., Zeng Y.D., Hu Z.X., Linag H. (2020). PCSK9 promotes the secretion of pro-inflammatory cytokines by macrophages to aggravate H/R-induced cardiomyocyte injury via activating NF-κB signalling. Gen. Physiol. Biophys..

[34] Ding Z., Pothineni N.V.K., Goel A., Lüscher T.F., Mehta J.L. (2020). PCSK9 and inflammation: Role of shear stress, pro-inflammatory cytokines, and LOX-1. Cardiovasc. Res..

[35] Andreadou I., Tsoumani M., Vilahur G., Ikonomidis I., Badimon L., Varga Z.V., Ferdinandy P., Schulz R. (2020). PCSK9 in Myocardial infarction and cardioprotection: Importance of lipid metabolism and inflammation. Front. Physiol..

[36] Gencer B., Mach F., Murphy S.A., De Ferrari G.M., Huber K., Lewis B.S., Ferreira J., Kurtz C.E., Wang H., Honarpour N. (2020). Efficacy of evolocumab on cardiovascular outcomes in patients with recent myocardial infarction: A prespecified secondary analysis from the FOURIER trial. JAMA Cardiol..

[37] White H.D., Steg P.G., Szarek M., Bhatt D.L., Bittner V.A., Diaz R., Edelberg J.M., Erglis A., Goodman S.G., Hanotin C. (2019). Effects of alirocumab on types of myocardial infarction: Insights from the OYSSEY OUTCOMES trial. Eur. Heart J..

[38] Stoekenbroek R.M., Lambert G., Cariou B., Hovingh G.K. (2018). Inhibiting PCSK9—Biology beyond LDL control. Nat. Rev. Endocrinol..

[39] Miura T., Kishioka Y., Wakamatsu J.-I., Hattori A., Nishimura T. (2010). Interaction between myostatin and extracellular matrix components. Anim. Sci. J..

[40] Speir R.W., Stallings J.D., Andrews J.M., Gelnett M.S., Brand T.C., Salgar S.K. (2015). Effects of valproic acid and dexamethasone administration on early bio-markers and gene expression profile in acute kidney ischemia-reperfusion injury in the rat. PLoS ONE.

[41] Verzola D., Cappuccino L., D’Amato E., Villaggio B., Gianiorio F., Mij M., Simonato A., Viazzi F., Salvidio G., Garibotto G. (2014). Enhanced glomerular Toll-like receptor 4 expression and signaling in patients with type 2 diabetic nephropathy and microalbuminuria. Kidney Int..

[42] Spallarossa P., Garibaldi S., Barisione C., Ghigliotti G., Altieri P., Tracchi I., Fabbi P., Barsotti A., Brunelli C. (2008). Postprandial serum induces apoptosis in endothelial cells: Role of polymorphonuclear-derived myeloperoxidase and metalloproteinase-9 activity. Atherosclerosis.

