# Supplementary material for: Renal Ischemia/Reperfusion Early Induces Myostatin and PCSK9 Expression in Rat Kidneys and HK-2 Cells
Source: Int J Mol Sci. 2021 Sep 13;22(18):9884. doi: 10.3390/ijms22189884 (PMC8465118; doi:10.3390/ijms22189884)
Supplement: Supplementary file 1 [file ijms-22-09884-s001.zip › ijms-1362582-supplementary.pdf]

Supplementary figures

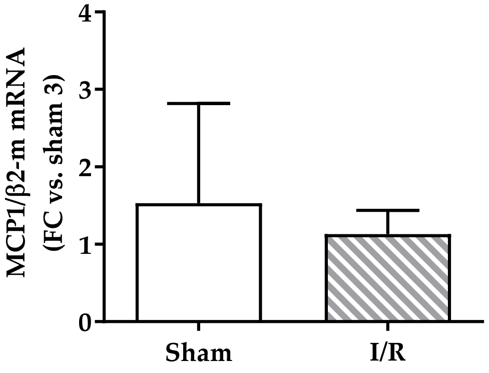

Figure S1. MCP-1 mRNA expression after 4 hours of reperfusion by quantitative real-time PCR. The results have been normalized by expressing the number of transcript copies as a ratio to β2-microglobulin (β2-m) and indicated as fold changes (FC) in respect to kidneys from sham-operated rat (#3).

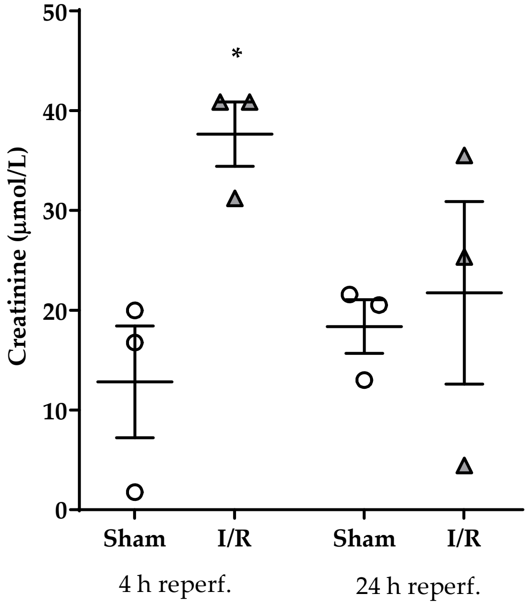

Figure S2. Plasma creatinine levels in rats undergone to sham intervention (circle) or I/R (triangle) after 4 h and 24 h of reflow; * $p < 0.05$.

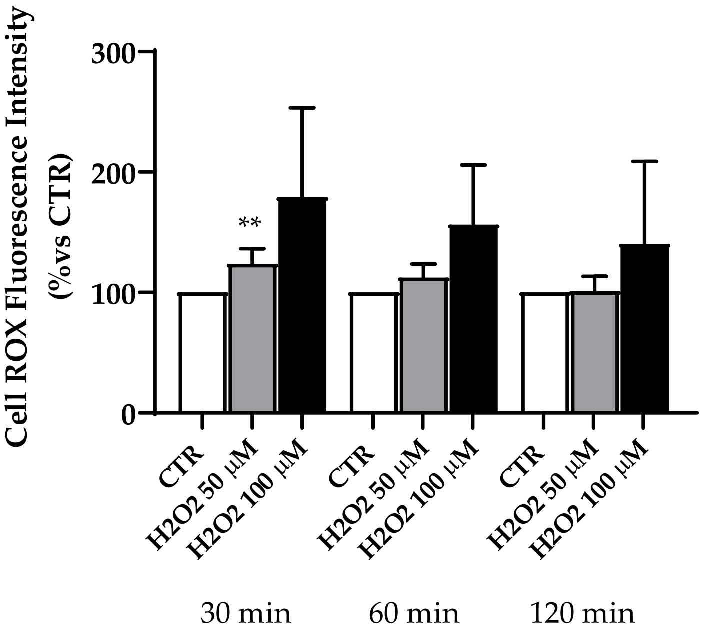

Figure S3. Quantification of cell ROS production by HK-2 cells after 30, 60, and 120 min of incubation with H_2O_2 using the CellROX™ kit (***p* < 0.01).

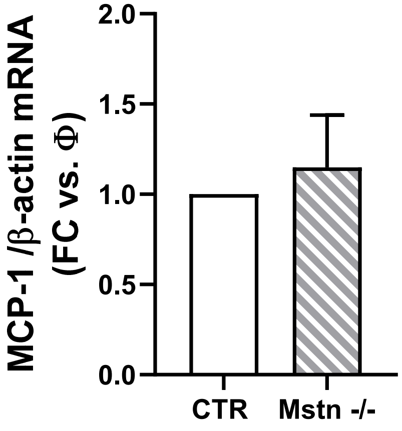

Figure S4. MCP-1 mRNA levels in Mstn siRNA cells after 3 hours of ischemia by quantitative real-time PCR. The results have been normalized by expressing the number of transcript copies as a ratio to β-actin and indicated as fold changes (FC) in respect to control siRNA cells.
